# Canine, Feline, and Murine Mammary Tumors as a Model for Translational Research in Breast Cancer

**DOI:** 10.3390/vetsci12020189

**Published:** 2025-02-19

**Authors:** Geovanni Dantas Cassali, Karen Yumi Ribeiro Nakagaki, Marisa Salvi, Marina Possa dos Reys, Marcos André Nino Rocha, Cecilia Bonolo de Campos, Enio Ferreira, Angelica Cavalheiro Bertagnolli Rodrigues, Diego Carlos dos Reis, Karine Araujo Damasceno, Alessandra Estrela-Lima

**Affiliations:** 1 Departamento de Patologia Geral, Instituto de Ciências Biológicas, Universidade Federal de Minas Gerais, Belo Horizonte 31270-901, Minas Gerais, Brazil; 2 Escola de Medicina Veterinária e Zootecnia, Universidade Federal da Bahia, Salvador 40170-110, Bahia, Brazil; 3 Princess Margaret Cancer Centre, University Health Network, Toronto, ON M5G 2M9, Canada; 4 Instituto de Pesquisas Veterinárias Desidério Finamor, Eldorado do Sul 92990-000, Rio Grande do Sul, Brazil; 5 Division of Molecular Pathology, The Institute of Cancer Research, London SW7 3RP, UK; 6 Centro de Pesquisas Gonçalo Moniz, Fiocruz, Salvador 40296-710, Bahia, Brazil

**Keywords:** mammary tumors, mammary cancer, histological grading, tumor microenvironment, translational models, cancer treatment

## Abstract

In veterinary medicine, mammary tumors are the most common neoplasms in female dogs and the third most frequent in cats, representing a significant challenge. Efforts have been directed toward adopting standardized diagnostic criteria to better understand tumor behavior and progression in these species. Meanwhile, the use of animal models has substantially advanced the understanding of comparative mammary carcinogenesis. These models provide critical insights into factors responsible for the disease in humans, with the expectation that such factors can be identified and controlled. In this context, this review presents a work based mainly on articles published by a research group specializing in mammary pathology (Laboratory of Comparative Pathology–Department of General Pathology–ICB/UFMG) and its collaborators, complementing their results with literature findings. The publications were categorized into animal research, experimental research, and human research. These studies addressed topics such as diagnosis, prognostic and predictive factors, tumor microenvironment, inflammation associated with tumors, treatment approaches, and factors influencing tumor growth. The conceptual network analysis underscores the importance of in vivo breast cancer models, both experimental and spontaneous, for understanding tumor progression mechanisms and therapeutic responses, offering valuable contributions to veterinary and human oncology.

## Simple Summary

Mammary tumors are a major health concern in female dogs and cats, requiring standardized diagnostic criteria for better management. Animal models have been crucial in advancing the understanding of mammary cancer, benefiting both veterinary and human medicine. This review summarizes research from a specialized group in mammary pathology, categorizing studies into animal, experimental, and human research. Key topics include diagnosis, prognostic and predictive factors, tumor microenvironment, inflammation, treatment strategies, and tumor growth. The findings highlight the essential role of in vivo cancer models in studying tumor progression and therapeutic responses, contributing to both veterinary and human oncology.

## 1. Introduction

A strategy that has significantly advanced the understanding of various aspects of comparative mammary carcinogenesis is the use of animal models [1,2,3]. For this purpose, transmissible tumors, transplantable tumors, and, more recently, genetically modified animals have been employed [1,3]. The goal of cancer research in animal models is to achieve a deeper understanding of the factors responsible for the disease in humans, with the expectation that these factors can be identified, eliminated, or controlled. Information on the occurrence of spontaneous tumors primarily originates from veterinary research on tumors in domestic animals, wildlife, and animals kept in zoos and laboratories. The study of the most frequent tumors in animals provides epidemiological data and insights into their etiology, as well as biological and therapeutic materials for research.

Companion animals and humans share remarkably similar living environments, exposing both species to analogous toxic substances, viruses, and/or pollution. These external stimuli contribute to the development of cancer in both species through genetic/epigenetic alterations, metabolic disruptions, and/or immune-system-related changes [4,5,6,7].

In this context, mammary tumors are the most frequent neoplasms in female dogs and the third most common in cats, representing a significant challenge in veterinary medicine. Substantial efforts have been directed toward the adoption of standardized diagnostic criteria, understanding tumor behavior and progression, and evaluating prognostic and predictive factors in these neoplasms. The knowledge and adoption of these parameters are essential for selecting effective therapies that reduce tumor recurrence and improve overall survival rates.

The first phase of our research prioritized the characterization and classification of mammary tumors, clinical staging, histological grading, and the standardization of prognostic and predictive markers in mammary gland samples from dogs, based on criteria used in human medicine. In the second phase, we initiated projects focused on studying the tumor microenvironment and treating mammary gland tumors in dogs. Until then, treatment had been almost exclusively based on surgical excision. We tested and introduced novel therapeutic alternatives, particularly for advanced cases where surgery alone is often ineffective, aiming to provide patients with prolonged survival and a good quality of life.

This review will discuss the work of a research group specializing in mammary pathology (Laboratory of Comparative Pathology–Department of General Pathology–ICB/UFMG) and its collaborations. To that end, this review will address the following topics: diagnosis, prognostic and predictive factors, tumor microenvironment, inflammation associated with tumors, treatment, and factors that influence tumor growth. This group primarily employs murine experimental models (Ehrlich Tumor, 4T1, CTag) and spontaneous models (dogs and cats) to study breast cancer. The initial studies with spontaneous models began in 1994 and have sought to apply the criteria and classifications used in human medicine to veterinary medicine, inspired by the One Health concept and the belief that, *“Between animal and human medicine, there are no dividing lines, nor should there be. The object is different, but the experience obtained forms the basis of all medicine.”–Rudolf Virchow.*

## 2. Conceptual Mapping of Thematic Connections in Breast Cancer Research

**Figure 1:**
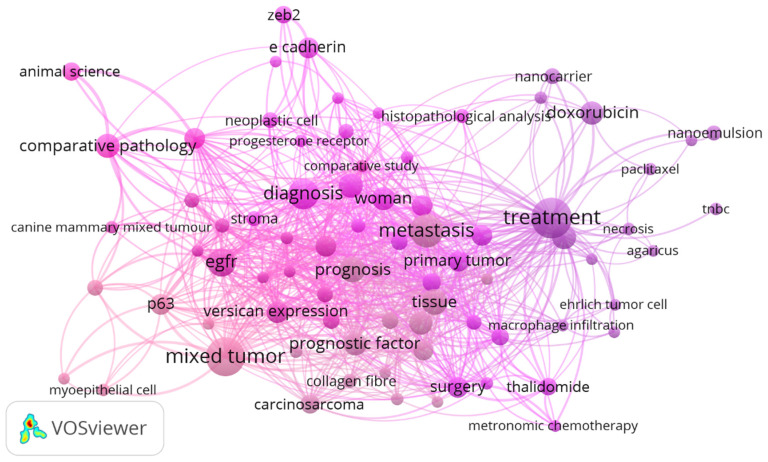
Interactive conceptual network of the main research themes in breast cancer, produced via bibliometric analysis of the articles published by the group.

The analysis of 174 articles published by the group led to the development of a comprehensive conceptual network based on 118 articles with a DOI. These articles were categorized into animal research (73 records), experimental research (44 records), and human research (1 record). Figure 1 illustrates this network, highlighting the main connections and emerging themes in breast cancer research.

Using the VosViewer software, we extracted bibliographic data to construct a network of concepts, which highlights the co-occurrences of relevant terms. VosViewer accesses the metadata of publications to identify and extract representative terms, forming a visual network that illustrates how these concepts interact. In this network, the sizes of the nodes indicate the levels of relevance of the terms, while the thicknesses of the lines represent the strengths of the interactions between the words.

The most prominent terms, such as mammary tumor, dog, mammary carcinoma, mouse, treatment, mixed tumor, human breast cancer, antitumor activity, comparative pathology, animal science, prognostic factors, veterinary medicine, histopathological analysis, and metastasis, reflect the predominant focuses of the scientific investigations (Figure 1). These terms demonstrate how different areas of study are interrelated, emphasizing the complex and interconnected structure of the group’s research.

The analysis of the conceptual network highlights the importance of in vivo breast cancer models, whether experimental or spontaneous, for understanding the mechanisms of tumor progression and therapeutic response. Continued advancement in this field is driven by interdisciplinary collaboration and translational research, integrating veterinary and medical oncology. This collaborative and integrated approach is essential to promote significant advances in breast cancer research.

## 3. Diagnosis

Mammary tumors in female dogs and cats exhibit epidemiological, clinical, biological, and genetic similarities to breast neoplasms in women and are thus considered important models in comparative studies [7,8,9,10,11,12,13]. Findings from our group demonstrate that the types of mammary tumors in humans, dogs, and cats are comparable, although the relative frequency of each tumor type differs [14,15,16].

The incidence of spontaneous mammary tumors in female dogs is two to three times higher than that observed in women, and, similar to human species, mammary tumors are rare in males [15,17]. The approximate percentage of malignant breast neoplasms in women is 15% [18,19], while in dogs and cats, it is around 85%, according to a recent survey carried out in Brazil (in which the total number of lesions evaluated in canines was 20,149 and in felines was 857) [20]. Regarding malignant tumors, a notable difference is observed: the most common carcinomas in women are invasive carcinomas of no special type, whereas in female dogs, the predominant types are carcinomas in mixed tumors, which, according to human classification, correspond to metaplastic breast carcinomas and carcinomas ex pleomorphic adenomas of the salivary gland [16,21,22]. Finally, when considering the frequency of other malignant neoplastic lesions of the mammary gland, such as sarcomas, it is evident that these are equally rare in both species [14,16].

In addition to the similarities between canine, feline, and human mammary neoplasms, several studies suggest the presence of preneoplastic changes in the mammary tissue of female dogs that are comparable to those observed in women. Epithelial hyperplasia and columnar cell changes are recognized in human breast tissue as the early stages of cellular transformation leading to breast cancer [23,24,25,26]. The presence of these intraepithelial changes in dogs and their association with mammary neoplastic lesions support the use of the canine species as a comparative model for studying non-invasive mammary lesions and their progression to breast cancer. According to the literature, preneoplastic lesions exhibit cytological and structural compositions similar to those observed in human mammary tissue. Consistent with these findings, our group describes atypical ductal hyperplasia and atypical columnar cell changes as lesions with morphological and immunophenotypic characteristics compatible with the process of neoplastic transformation [24].

**Figure 2:**
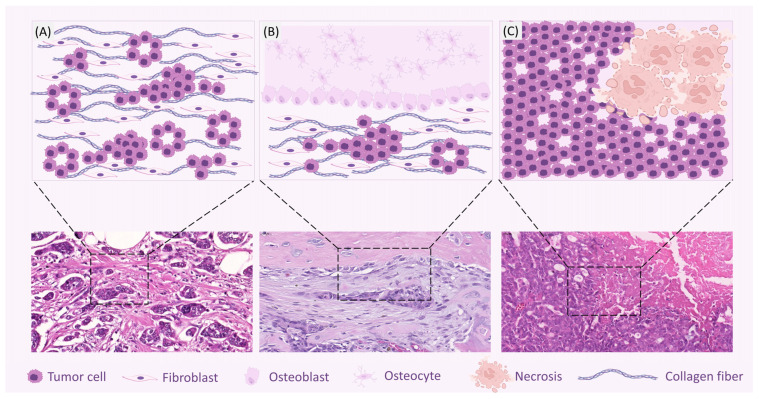
Graphic representations and photomicrographs of the most frequent histological types in humans, canines, and felines. (**A**). Invasive carcinoma (human). (**B**). Carcinoma in a mixed tumor (canine). (**C**). Cribriform carcinoma (feline). Created with BioRender.com (accessed on 16 February 2025).

Several histological subtypes described in women have already been identified in female dogs and cats (Table 1 and Figure 2), including invasive micropapillary carcinoma, secretory carcinoma, and pleomorphic lobular carcinoma, among others [27,28,29,30,31]. Invasive micropapillary carcinoma, although rare in both humans and dogs, exhibits distinct aggressive behavior and a poor prognosis. It has been observed that the clinicopathological and immunophenotypic characteristics, as well as overall survival, are similar between dogs and women. This subtype is characterized by aggressive behavior, high rates of metastasis to regional lymph nodes, and short overall survival [32]. As in humans, canine mammary cancer usually affects older individuals, with the average age being around 10 years, rarely occurring before 5 years of age [33] and, when using the Lebeau table, it appears that it occurs in the corresponding age groups [34].

Pleomorphic lobular carcinoma of the breast is a recognized subtype of invasive lobular carcinoma in humans. A significantly worse prognosis and survival rate are reported for the pleomorphic subtype. In female dogs, the same histological pattern, immunophenotypic characteristics, and aggressive behavior, including frequent metastasis to lymph nodes, have been identified [28].

Secretory carcinoma is a rare histological type first described in female dogs in 1999, with few subsequent reports [27,35]. In women, it is described as an uncommon variant of breast cancer, characterized by intracellular and extracellular eosinophilic secretions. Immunohistochemical findings in female dogs and cats are similar to those described for secretory carcinoma in humans, including positive staining for cytokeratin and lactalbumin [36]. In contrast to human patients, where this histological type exhibits limited aggressiveness, particularly in young patients, in dogs, it demonstrates aggressive behavior, with both regional and distant metastases [27,37].

In human medicine, basaloid carcinoma is described in the salivary gland and is morphologically similar to its benign counterpart, the basaloid adenoma [36]. In female dogs and cats, basaloid adenomas and carcinomas have been described in the mammary glands, exhibiting histological characteristics similar to those of human salivary glands [14,37,38,39]. The malignant variant of this histological type appears to display aggressive behavior, with shorter survival times [38].

In our diagnostic routine, benign mixed tumors and carcinomas in mixed tumors are the most frequently diagnosed histological types in the mammary glands of female dogs and less frequently in cats. Together with carcinosarcomas and sarcomas in mixed tumors, they form a group characterized by a combination of epithelial (luminal and myoepithelial) and mesenchymal components with a variable matrix, which may be myxoid, chondroid, or osseous [16]. According to a survey of mammary lesions diagnosed over 15 years at the Laboratory of Pathology, Federal University of Minas Gerais (UFMG), Brazil, mixed tumors account for 60% of the mammary neoplasms diagnosed [16].

Histologically, benign mixed tumors are well-demarcated, composed of epithelial cell proliferation within a myxoid matrix with ectopic cartilage and bone formation. The epithelial cells are cuboidal or columnar, arranged in tubular or papillary patterns, and are lined by a layer of myoepithelial cells. The myxoid stroma contains spindle-shaped and stellate myoepithelial cells. Cartilage appears as nodules or plaques of variable size, with well-differentiated chondroblasts and chondrocytes. Osseous tissue consists of osteoblasts producing osteoid and mineralized bone [40,41,42,43,44].

Carcinomas in mixed tumors are characterized by carcinomatous proliferation, which can be in situ, microinvasive, or invasive, within benign mixed tumors. The carcinomatous proliferation may exhibit tubular, papillary, or micropapillary arrangements, or clusters and nests of cuboidal or columnar epithelial cells. Malignant epithelial cells are pleomorphic with atypical mitoses. Carcinosarcomas are composed of carcinomatous and sarcomatous proliferations. As observed in carcinomas in mixed tumors, the carcinomatous component may present in situ, microinvasive, or invasive proliferation with variable epithelial arrangements. The sarcomatous component consists of pleomorphic to polygonal cells resembling fibroblasts, osteocytes, and other mesenchymal tissues such as chondroid, adipose, and vascular tissue, exhibiting high cellularity, nuclear atypia, and an elevated mitotic index [42,43].

Given the high prevalence of mixed tumors and the heterogeneity of histological patterns, which can complicate diagnosis, our group has focused on standardizing morphological criteria and expanding knowledge about the histogenesis, malignant progression, and prognosis of these neoplasms.

In humans, benign mixed mammary tumors are rare; however, they are common neoplasms in the salivary glands, where they are referred to as pleomorphic adenomas [45]. In both cases, the epithelial component of these tumors can undergo malignant transformation, resulting in carcinomas in mixed tumors (in female dogs) or carcinomas ex pleomorphic adenomas (in human salivary glands) [43,46,47].

A comparative analysis between canine mammary mixed tumors and human salivary gland tumors revealed that both exhibit similar clinical, histological, and antigenic characteristics [48]. Another study found that the proportion of neoplasms with a luminal immunophenotype was comparable between carcinomas ex pleomorphic adenomas and carcinomas in mixed tumors [49]. These similarities highlight the applicability of canine mammary mixed tumors as comparative models for studying malignant transformation.

In general, female dogs with carcinomas in mixed tumors exhibit longer survival compared to those with other types of carcinomas [50,51]. Conversely, female dogs with carcinosarcomas typically have a poorer prognosis compared to other types of mammary neoplasms due to high rates of nodal and distant metastasis [50,51,52].

In a study conducted by our group that evaluated 162 benign mixed tumors, 682 carcinomas in mixed tumors, and 60 carcinosarcomas, it was observed that female dogs with benign mixed tumors presented smaller lesions (T1 and T2) compared to those with carcinosarcomas. Female dogs with benign mixed tumors were younger than those with carcinomas in mixed tumors or carcinosarcomas. The proportion of nodal or distant metastases was higher among dogs with carcinosarcomas compared to those with carcinomas in mixed tumors [52].

Interestingly, carcinomas in mixed tumors with micropapillary or solid proliferation patterns were associated with shorter survival compared to other proliferation patterns [52]. These findings demonstrate that carcinomatous areas in mixed tumors with more aggressive histological patterns can influence the behavior of these neoplasms. Therefore, a thorough evaluation of carcinoma in mixed tumors is essential to establish the prognosis. This is particularly important because female dogs with carcinomas in mixed tumors that exhibit areas of more aggressive histological types may benefit from adjuvant chemotherapy [16,52,53].

## 4. Prognostic and Predictive Factors

In mammary tumors in dogs and cats, as in breast cancer in women, several clinical and pathological factors, such as tumor size, lymph node involvement, histological type, and grade, are evaluated to establish the clinical staging and determine the prognosis [4,54,55,56,57,58]. Additionally, molecular markers are also assessed as sources of information to predict the prognosis in various types of cancers in female dogs, cats, and women [59,60,61]. In Table 2, we present the prognostic and predictive factors for canine, feline, and human mammary neoplasms.

The stage of mammary gland cancer is an important prognostic factor applied in women as well as in female dogs and cats. It is determined according to the TNM classification (primary tumor size, regional lymph node involvement, and distant metastasis). All individual characteristics of the TNM classification carry prognostic significance [21,54,57,63].

Tumor size, based on tumor diameter, is one of the most established factors for evaluating the breast cancer prognosis in women, as well as in female dogs and cats. Numerous studies confirm a correlation with shorter disease-free survival and overall survival in female dogs with large tumors (>5.0 cm) [16,64]. However, it has been observed that even tumors smaller than 1.0 cm can be malignant and present metastasis at the time of diagnosis.

The definition of the histological type in malignant tumors, particularly mammary carcinomas, can be considered a prognostic parameter. Poorly differentiated mammary carcinomas, forming solid masses or even exhibiting organizational patterns distinct from normal mammary tissue, tend to have a poor or guarded prognosis [41].

In female dogs and cats, certain solid tumors, micropapillary carcinomas, and carcinosarcomas are recognized as aggressive tumors with a higher likelihood of metastasis development, and therefore a worse prognosis when considering the disease-free time and overall survival [16,37].

In human medicine, the most widely used histological grading system for invasive carcinomas is the Nottingham system, which corresponds to the Scarff, Bloom, and Richardson (SBR) grading system [65], modified by Elston and Ellis (1998) [66]. These authors introduced the importance of the field diameter in assessing the mitotic index. In this system, the histological grade is based on the evaluation of tubular formation, nuclear pleomorphism, and mitotic count. The histological grade has been considered an independent prognostic indicator in human invasive breast cancer. A positive and significant correlation is observed between histological differentiation and the prognosis. Patients with poorly differentiated tumors (Grade III) have a shorter survival time compared to those with moderately (Grade II) and well-differentiated (Grade I) carcinomas [66]. The adaptation of this system for invasive carcinomas in female dogs and cats has shown results similar to those observed in humans [16,67,68,69,70,71,72].

The neoplastic involvement of regional lymph nodes is associated with a worse prognosis, as canine, feline, and human patients with lymph node involvement have a shorter survival time compared to those without lymph node metastasis [14,16,55,68,73,74]. Similar to human breast cancer, a higher number of affected lymph nodes is also associated with a shorter survival time in female dogs and cats. However, there are few studies that analyze the sizes of metastases in lymph nodes, extracapsular extension, and their correlation with survival in female dogs [14,68,75].

Although the majority of mammary pathology diagnosis is performed using hematoxylin- and eosin-stained sections, some cases require immunohistochemistry for a proper evaluation [54]. Molecular analysis in human oncology has been explored for decades, primarily aiming to establish diagnostic, prognostic, and predictive markers or to understand the mechanisms involved in carcinogenesis and breast cancer progression [76,77,78,79,80,81,82]. In veterinary medicine, this analysis is more recent and has been developed based on what is recommended for human medicine [82,83,84,85,86,87,88].

Immunohistochemical markers allow the determination of the presence of hormone receptors (estrogen receptor, progesterone receptor), the cell proliferation rate (Ki-67), angiogenesis (VEGF, CD31, CD34), and pro-angiogenic determinants such as the COX-2 enzyme, as well as the evaluation of markers indicative of tumor aggressiveness, such as the expression of receptors for epidermal growth factors (EGFR and HER-2) and transcriptional markers of proliferation and epithelial–mesenchymal transition (c-Kit, cadherins, CD44, ZEB1 and 2, TWIST) in female dogs [89].

In human breast cancer, the greatest challenge in determining the prognosis is the heterogeneity of this type of neoplasia. Tumors with similar histological types, clinical stages, and differentiation grades may have different prognoses and therapeutic responses [90]. For this reason, different technologies have been used to stratify breast cancer types based on molecular similarities. Similarly, in female dogs and cats, a possible immunohistochemical classification of mammary carcinomas, referred to as “immunophenotypes (molecular profile)”, has been proposed. Based on this, carcinomas are categorized into five groups: luminal A, luminal B HER-2 negative, luminal B HER-2 positive, HER-2 overexpressed, and triple-negative/basal-like (Table 3). These tumors, classified into immunophenotypes, exhibit distinct clinical characteristics, and in addition to helping resolve tumor heterogeneity, they can infer the behavior and therapeutic response of the animals [62,90,91].

Estrogen and progesterone play a crucial role in mammary gland development, lactation, and tumorigenesis. Studies have demonstrated variations in the expression of receptors for these hormones during the development of mammary neoplasms. These variations seemingly have implications for patient prognosis in both dogs and cats [89]. Higher expression of these markers is observed in benign tumors compared to malignant ones [92]. In both species, lower estrogen receptor expression is found in poorly differentiated mammary carcinomas or more aggressive histological subtypes. In dogs, a relationship between lower estrogen expression, larger tumor masses, and shorter survival times has been described [92]. The evaluation of the combined expression of both hormone receptors, estrogen and progesterone, has been shown to better define the prognostic behavior of the patient. Tumors negative for both receptors have a worse prognosis compared to those positive for both or only one receptor.

In cats, most tumors exhibit high expression of estrogen and progesterone receptors; however, this expression does not correlate with aggressive histological parameters or overall survival after a mammary gland cancer diagnosis. This high hormonal expression, according to some authors, represents a potential avenue for future studies on hormone therapy [48].

The evaluation of cellular proliferative activity through immunohistochemical markers is recognized as an important prognostic parameter for mammary lesions in humans, dogs, and cats [40,48,59]. Higher immunostaining for Ki-67 is observed in malignant mammary neoplasms and is correlated with lower hormonal receptor expression, a higher tumor histological grade, a larger tumor size, the presence of metastases, and shorter survival times in both female dogs and cats [59,93].

HER2 (Human Epidermal Growth Factor Receptor 2) is a surface protein involved in cell growth. In breast cancer, overexpression of this protein (usually due to HER2 gene amplification) can occur and is associated with a more aggressive form of the disease [21]. The importance of HER2 as a prognostic and predictive factor in breast cancer is significant, as it helps guide treatment decisions and better understand tumor behavior [55]. In dogs and cats, HER2 is also considered an important tumor marker, regulating tumor growth, survival, and differentiation. In dogs, a positive correlation has been reported between HER2 expression and a high mitotic index, high histological grade, and the tumor size, although no relationship with survival has been identified [69].

In feline mammary carcinomas, HER2 expression is well-studied. HER2 in cats shows high homology with the human protein and is associated with increased cellular proliferation, the formation of large neoplastic masses, higher histological grades, and gene amplification of HER2. These findings support the idea that HER2 expression analysis may be considered a relevant prognostic factor [57].

Cyclooxygenases are enzymes that catalyze the conversion of arachidonic acid into a variety of prostaglandins. There are two cyclooxygenase isoforms, COX-1 and COX-2. COX-2 is the isoform most frequently studied in relation to neoplastic development and progression, as it is directly linked to tumor angiogenesis. Increased immunohistochemical expression of COX-2 is associated with unfavorable prognostic factors such as a larger tumor size, more aggressive histological subtypes, higher metastatic potential to lymph nodes, and high histological grade. COX-2 is also considered an important predictive factor in canine and feline mammary carcinomas [57,94]. A recent comparative study on micropapillary carcinomas in dogs and women revealed similar patterns of COX-2 expression between the two species [95].

Tumor markers are substances present in tumors, the blood, or other biological fluids, primarily produced by neoplastic cells or normal tissue in response to the presence of the tumor. Serum tumor markers, which until recently were primarily studied in human neoplasms, have gained significant importance in veterinary medicine. The serum markers CEA, CA15.3, and LDH have been studied in dogs with and without mammary neoplasms and were measurable in all groups (healthy, with metastases, and without metastases in lymph nodes). Higher serum concentrations of CA15.3 and LDH were associated with regional and distant metastases. A significantly higher serum concentration of CA15.3 was found in animals with lymph node metastases compared to those without metastases. No significant differences in CEA levels were observed between groups [96].

## 5. Tumoral Microenvironment

Recent studies have highlighted that breast cancer is not solely composed of neoplastic cells but also involves significant modifications in the surrounding stroma, known as the tumor microenvironment. These changes play a pivotal role in the development and progression of breast cancer and present potential opportunities for therapeutic intervention. Key components of the tumor microenvironment, such as immunosuppressive cells, soluble factors, and an altered extracellular matrix, collaborate to suppress antitumor immune responses and drive tumor progression and metastasis. The stromal cells within the breast cancer microenvironment undergo molecular changes and exhibit abnormal signaling pathways, some of which are valuable predictors of clinical outcomes. The tumor microenvironment (TME) has gained significant attention in research due to its complex cellular and non-cellular composition, which can profoundly influence the diagnosis, prognosis, and treatment of breast cancer patients [97,98,99].

### 5.1. Preclinical Studies In Vitro and in Animal Models

Among the research lines developed by the group, preclinical studies on the tumor microenvironment in experimental models have made significant contributions to oncology.

One study focused on tumor cell heterogeneity, a critical factor in determining therapy responses and tumor progression, emphasizing the importance of molecular profiling to identify therapeutic targets and vulnerabilities in cancer treatment. The research evaluated cellular heterogeneity in triple-negative breast cancer (TNBC) cell lines BT-549 and Hs 578T, cultured in monolayer and tumor sphere models. Fluorescence and electron microscopy, along with flow cytometry analysis, revealed substantial morphological and phenotypic variation both within and between these cell lines, characterized by mesenchymal traits such as the absence of E-cadherin and the formation of filopodia [100].

Additionally, the transcription factor SOX3 was found to play a pivotal role in regulating epithelial markers, promoting E-cadherin (ECAD) expression while suppressing N-cadherin (NCAD), SNAIL, ZEB1, and ZEB2, thereby maintaining epithelial characteristics in breast cancer cells. SOX3 also enhanced apoptosis in MDA-MB-231 cells, with its low expression associated with reduced apoptosis in ductal carcinomas. These findings highlight the tumor-suppressive potential of SOX3, warranting further investigation [101].

A robust line of research has focused on exploring the diverse factors influencing cancer progression using experimental models such as Ehrlich tumors and the murine mammary carcinoma 4T1. These studies aim to contribute to the understanding of the tumor microenvironment, progression, metastasis, and the development of antineoplastic therapies.

The 4T1 murine mammary carcinoma is a model of triple-negative breast cancer that exhibits rapid and progressive metastatic development when orthotopically and heterotopically inoculated in BALB/c mice [96]. The metastases in this model display a distribution similar to that of human and canine mammary glands, with the lungs, liver, bones, and brain being the main metastatic sites. Furthermore, tumor development in this model leads to significant changes in both the primary and metastatic tumor microenvironments, with a notable accumulation of various immune response cells associated with alterations in extracellular matrix components and angiogenesis [97,98].

In addition to the 4T1 murine mammary carcinoma, we also extensively use the Ehrlich tumor for experimental therapeutic studies [49,102,103,104,105,106,107,108,109]. The Ehrlich tumor is a transplantable tumor originating from the mammary glands of female mice, which grows in an ascitic form when inoculated into the peritoneum or in a solid form, typically inoculated into the mammary gland, flank, and paws [108,110].

The influence of allergic conditions on the progression of Ehrlich tumors was also studied. The research showed that chronic antigen ingestion by sensitized mice resulted in reduced tumor progression, mediated by increased cellular apoptosis and reduced necrotic areas. These findings suggest a potential protective mechanism in allergic individuals [109].

The research also evaluated the efficacy of the recombinant protein Kint3-4 in inhibiting tumor growth and angiogenesis in Ehrlich tumors. Mice treated with Kint3-4 exhibited reduced tumor growth, decreased inflammation, and a lower microvessel density, demonstrating a significant antiproliferative effect compared to the control group [103]. Additionally, the expression of Qa-2 in solid Ehrlich tumors and its relationship with the profile of infiltrating lymphocytes were analyzed. It was observed that Qa-2 expression increased during intermediate stages of tumor development and continued to rise in later stages, correlating with an increase in CD3+ cells. This finding suggests that Qa-2 may play a relevant immunomodulatory role in tumor progression [108].

The studies conducted by this research group have significantly advanced the understanding of the molecular and cellular mechanisms associated with breast cancer, utilizing both the Ehrlich tumor and the murine 4T1 model. A noteworthy focus was the evaluation of the antineoplastic effects of thalidomide. The results demonstrated thalidomide’s remarkable capacity to reduce tumor growth and pulmonary metastasis in this model. Thalidomide exerted its effects by suppressing critical processes such as angiogenesis and inflammation, evidenced by reduced blood vessel formation and lower levels of pro-inflammatory factors [110].

In addition, the effects of thalidomide on 4T1 mammary carcinoma were evaluated from a hematological perspective. The therapy led to a significant increase in circulating leukocyte counts, which, interestingly, coincided with reduced tumor growth, suggesting a possible link between immune response activation and tumor inhibition. Another aspect explored was the immunomodulatory role of thalidomide, particularly concerning tumor-associated macrophages (TAMs). The treatment not only reduced primary tumor size and pulmonary metastasis but also influenced TAM infiltration, indicating that different doses of thalidomide may variably modulate the tumor microenvironment [111].

### 5.2. Studies in Canine Models

Research involving canine models has provided valuable insights into the tumor microenvironment, cancer progression, and therapeutic interventions, with a focus on translational applications to human oncology. One line of investigation explored the molecular and cellular characteristics of canine mammary tumors, particularly mixed mammary tumors (CMTs). These studies highlighted the role of the extracellular matrix, immune infiltration, and stromal composition in tumor behavior.

### 5.3. Tumor Stroma

In the pursuit of a better understanding of the tumor microenvironment, the stroma and its components have been extensively studied. The extracellular matrix is a key element of the tumor microenvironment. Beyond providing structural support for the growth of solid tumors, it is directly involved in cellular proliferation, differentiation, migration, and tumor progression.

In this context, the characterization of mesenchymal components in canine mixed mammary tumors has been a focus of research. Auler et al. (2011) [112] evaluated benign mixed tumors and carcinomas within mixed tumors (CMTs) and observed that myeloid metaplasia is associated with the presence of blood capillaries and megakaryocytes but is not linked to the benign or malignant nature of the tumors.

The components of the extracellular matrix have also been studied to elucidate the mechanisms underlying the biological behavior of tumors. Damasceno et al. (2012) [113] were the first to investigate the expression of versican in in situ and invasive carcinomatous regions of canine CMTs and to evaluate potential associations between versican expression, classical prognostic factors, and overall survival. In this study, the confirmation of invasive areas was conducted by identifying the loss of staining for p63 and smooth muscle α-actin (α-SMA) in canine CMT cases. Versican immunoreactivity was found to be less intense in areas adjacent to in situ carcinomatous regions compared to invasive regions, which exhibited extensive and strong staining, suggesting a direct relationship between versican and tumor invasiveness.

The expression of versican and its relationship with p63 and α-SMA were further evaluated in myoepithelial cells at different stages of differentiation in canine mixed mammary tumors [114]. A significant variation in versican expression was observed across different stages of myoepithelial cell differentiation, with an inverse correlation between versican and p63/α-SMA expression. Specifically, spindle-shaped or stellate myoepithelial cells producing myxoid matrix showed lower p63/α-SMA expression and higher versican expression, indicating that dedifferentiation was associated with increased production of this proteoglycan in the matrix.

The critical role of myoepithelial cells in versican production within mixed tumors was further demonstrated by Figueiredo et al. (2021) [115]. This study revealed that versican expression was similarly associated with invasiveness in carcinomas with and without myoepithelial proliferation. However, higher levels of versican were observed in CMTs, suggesting that the presence of myoepithelial cells in this tumor type contributes to the stromal composition and promotes increased versican expression.

We then sought to understand the relationship between the proteoglycan versican and the receptors CD44 and the EGFR family, which are expressed on the membrane of carcinomatous cells and identified as key mediators linking versican to tumor cells [116]. The study suggests that the coexpression of versican with these receptors is associated with more aggressive characteristics of mammary tumors in female dogs, indicating that versican may play a significant role in tumor progression. The analysis of these proteins provided valuable insights for the prognosis and treatment of these tumors.

This relationship between versican and EGFR/HER-2 was further explored by Damasceno et al. (2016) [117]. In this investigation, it was demonstrated that tumors with low versican expression exhibited higher EGFR staining in in situ areas, whereas tumors with high versican expression displayed increased staining for both EGFR and HER-2 in in situ areas. These findings suggest that these molecules may play a critical role during the early stages of tumor progression.

Rodrigues and collaborators (2016) [118] investigated the subcellular localization of epidermal growth factor receptor (EGFR) in invasive micropapillary carcinoma (IMPC) of the canine mammary gland using confocal immunofluorescence microscopy. Their study revealed that EGFR co-localized with the inner nuclear envelope marker Lamin B1 in this tumor, suggesting that EGFR localization may serve as a predictive biomarker for a therapeutic response in IMPC. Subsequently, these authors [119] characterized the neoplastic cells outlining the cystic spaces of IMPC in canine mammary glands using immunohistochemistry (IHC), immunofluorescence (IF), super-resolution microscopy, and transmission electron microscopy (TEM). Their analysis provided insights into the cellular composition of the cystic areas. The loss of epithelial cell polarity in IMPC was demonstrated by the mislocalization of MUC1 to the stroma-facing surface. Additionally, the cystic spaces were shown to be lined by myoepithelial-like cells that had lost p63 expression. These findings contribute to a deeper understanding of the cellular microenvironment in invasive tumors, offering potential advancements in cancer diagnosis and treatment.

Second harmonic generation and multiphoton excitation fluorescence microscopy were utilized to evaluate collagen fiber alterations in human and canine mammary neoplasms [120]. The analysis demonstrated a lower density of collagen fibers and a higher density of cells in both human and canine mammary carcinomas compared to normal tissue, with collagen fibers being more aligned in tumors. Shorter collagen fibers were observed in carcinomas with an unfavorable prognosis, correlating with clinical and pathological data in both species. Subsequently, we investigated collagen modifications that predict lymphatic metastasis in dogs with carcinomas in mixed mammary tumors. Carcinomas with lymph node metastasis (CMTMs) exhibited shorter, more wavy collagen fibers compared to carcinomas without lymph node metastasis (CMTs), underscoring the critical role of collagen in invasion and the dissemination of lymphatic metastasis [121].

### 5.4. Tumor-Associated Inflammation

Mammary neoplasms in women, dogs, and cats are characterized by the infiltration of a variety of immune cells, which play critical roles in both promoting and inhibiting tumor growth, significantly impacting prognosis [122,123,124]. In human breast cancer, the tumor microenvironment (TME) contains cells that can determine the prognosis. CD8+ T cells, natural killer (NK) cells, M1 macrophages, and dendritic cells are associated with favorable outcomes as they secrete tumor-inhibiting factors and cytokines that stimulate the immune system, inhibiting angiogenesis and tumor metastasis [125,126,127,128].

Conversely, cells such as M2 macrophages, cancer-associated fibroblasts (CAFs), myeloid-derived suppressor cells (MDSCs), tumor-associated endothelial cells (TECs), pericytes, mesenchymal stem cells (MSCs), and cancer-associated adipocytes (CAAs) contribute to an environment that favors tumor progression [129,130,131,132,133,134,135,136]. Additionally, there are cellular compositions whose predictive role is complex and not yet fully elucidated, sometimes promoting while other times inhibiting neoplastic growth, such as regulatory T cells (Tregs), B cells, CD4+ T cells, and tumor-associated neutrophils (TANs) [135,136,137,138].

The majority of infiltrating leukocytes in breast tumors are lymphocytes [121]. Studies indicate that intense lymphocytic infiltrates correlate with favorable prognoses, especially in molecular subtypes like triple-negative (TNBC) and HER2+ [139]. Recent studies suggest that tumor-infiltrating lymphocytes (TILs) are associated with responses to cytotoxic treatments and immunotherapy, particularly for patients with triple-negative breast cancer [140]. However, the presence of lymphocyte subpopulations such as Tregs can indicate unfavorable prognoses due to their ability to suppress antitumor responses.

Malignant mammary tumors in dogs are also characterized by significant infiltration of mononuclear cells, particularly lymphocytes, whereas lymphocytes are rarely observed in normal mammary glands and benign mammary tumors [123]. High-grade tumors with lymphatic invasion exhibit greater infiltration by Tregs, which is associated with a poorer prognosis, lower survival, and more aggressive disease characteristics [141].

Regarding macrophage infiltration in mammary tumors, in both human and canine patients, their increased presence without distinction in the activation profile can be related to better clinical staging, grading, higher expression of class I MHC molecules in circulating monocytes, and overall survival. However, a significant presence of M2 macrophages is related to more aggressive clinicopathological characteristics, such as lymph node metastasis, a shorter survival time, and a poorer prognosis. In mammary carcinomas of women and dogs, inflammatory infiltrates that relate to a better response to chemotherapy show a predominance of pro-inflammatory (M1) macrophages, whereas poorer survival is associated with anti-inflammatory (M2) macrophages.

Similar to mammary tumors in women and dogs, in feline mammary carcinomas, CD3+ T lymphocytes represent the main immune-infiltrating subpopulation. High infiltration of CD3+ T cells combined with CD8+ T lymphocytes and tumor-associated macrophages (TAMs) of the M1 subtype suggest better outcomes in cats with triple-negative tumors [97]. CD8+ T cells constitute a new favorable prognostic factor. B lymphocytes constitute a significant portion of the lymphocyte population, and along with plasma cells, are observed more frequently in smaller tumors without lymphatic invasion or lower grading, indicating a relationship with the biological behavior of the tumor and suggesting a possible protective effect of these cells or their presence in the early phase of tumor development [97]. On the other hand, regulatory T cells (Tregs) and tumor-associated macrophages (TAMs) of the M2 subtype have been linked to immunosuppression, tumor progression, undifferentiated tumors, more aggressive behavior, promotion of angiogenesis, modulation of the extracellular matrix, and poorer disease-free survival (DFS) and overall survival (OS) rates [97,124,142]. Additionally, elevated neutrophil-to-lymphocyte ratio (NLR) levels have been correlated with unfavorable outcomes, suggesting they are an important indicator of the intensity of the inflammatory process [143].

In experimental models using rodents, primary tumors have been observed to activate “tumor-entrained neutrophils”, which may inhibit the targeting of metastases to the lungs [144] and can reduce the proliferation of CD8+ T lymphocytes; however, their role in human and veterinary medicine remains uncertain [135].

Recognizing cellular heterogeneity in the tumor microenvironment can guide personalized therapeutic strategies, enhancing treatment efficacy and improving the prognosis in both humans and animals. To improve understanding of the role of immune infiltration in mammary tumors, our group has conducted several studies with dogs over the years. In 2010, Estrela-Lima et al. [145] evaluated the immunophenotypic profile of infiltrating lymphocytes in canine mammary carcinomas, aiming to associate them with prognostic factors and survival rates. It was concluded that analyzing this profile can offer a valuable tool for predicting the prognosis in dogs with mammary carcinoma. The intensity of lymphocytic infiltration and the balance between CD4+ and CD8+ T lymphocytes emerge as potential prognostic biomarkers that can guide clinical and therapeutic decisions. These findings underscore the importance of considering the tumor’s immunological microenvironment in managing canine mammary gland cancer, which holds promise for similar research in human breast cancer.

In 2018, Souza et al. [146] investigated the density of inflammatory cells and the percentage of tumor fibrosis in canine mammary neoplasms, relating these parameters to different histological types. It was found that the composition of the inflammatory infiltrate and the presence of tumor fibrosis are critical factors in determining the aggressiveness of mammary tumors. A high density of neutrophils and plasma cells may indicate an unfavorable prognosis, while the presence of macrophages and CD8+ T lymphocytes is associated with a more positive prognosis.

Also in 2018, Vieira-Filho et al. [147] evaluated the density of tumor-associated macrophages (TAMs) in the mammary carcinoma microenvironment and the expression of SOCS1 and SOCS3 proteins. It was concluded that the expression of SOCS3 in activated macrophages is associated with an effective antitumor immune response, better clinical parameters, and higher survival rates. In contrast, activation related to SOCS1 was associated with tumor progression and reduced survival rates. These results suggest that SOCS proteins play a role in modulating the immune response in the tumor microenvironment, directly influencing the prognosis of mammary neoplasms.

In the same year, Monteiro et al. [148] studied the relationship of TAMs with tumor progression and macrophage polarization into M1 and M2 subtypes in canine mammary tumors. The study concluded that TAMs play an important role in the progression and aggressiveness of these tumors. Macrophage polarization to the M2 phenotype is associated with a more permissive tumor microenvironment, favoring tumor growth and invasion. These findings suggest that TAMs, especially the M2 subtype, may serve as therapeutic targets to improve the prognosis in dogs with breast cancer.

In 2022, Damasceno and colleagues [149] analyzed immune evasion mechanisms in dogs with inflammatory mammary carcinoma. It was concluded that inflammatory mammary carcinoma is associated with an altered immune response, characterized by intense lymphocytic infiltration and increased but ineffective cytotoxic activity. Low expression of FAS-L and a high presence of macrophages contribute to immune evasion and aggressive tumor progression.

Recently, Flecher et al. [150] characterized the immunophenotype and degree of inflammation in solid-type canine mammary neoplasms, investigating their association with metastasis, the Ki-67 index, tumor size, necrosis, and survival. The study concluded that the immunophenotype and inflammation level are critical factors in determining the prognosis of mammary neoplasms. As expected, neoplasms with a triple-negative immunophenotype exhibited greater aggressiveness and shorter survival times. These findings suggest that a detailed assessment of the immunological and inflammatory profile is essential for the clinical and therapeutic management of mammary neoplasms, contributing to a more personalized and effective approach to combating breast cancer.

**Figure 3:**
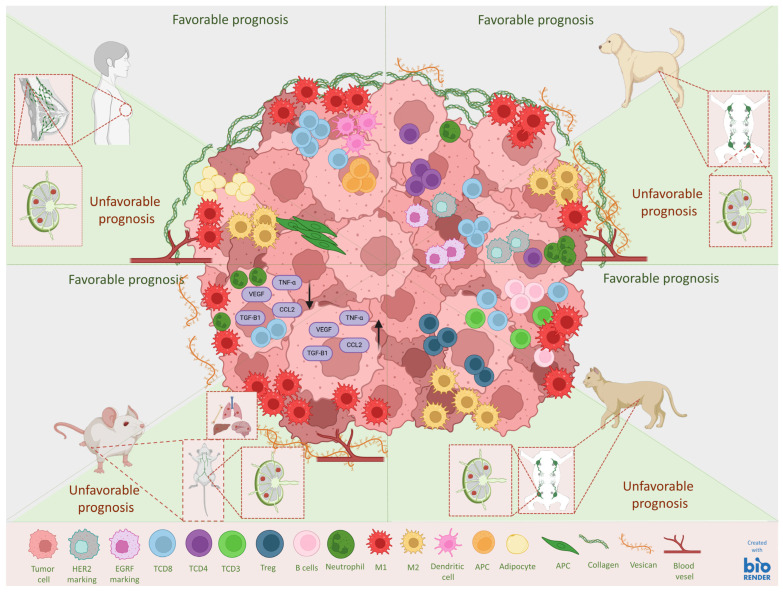
Graphical representation of the favorable and unfavorable prognostic aspects of the tumoral microenvironment in humans, canines, felines, and mice, representing the anatomical aspects of the mammary gland and regional lymph nodes.

The favorable and unfavorable prognostic aspects of the tumoral microenvironment in the described species are summarized in Figure 3.

## 6. Treatment and Factors Influencing Tumor Growth

The study of cancer biology has provided numerous possibilities for diagnosis and therapeutic strategies while simultaneously revealing its enormous complexity, which impairs the clinical success of many therapies. These challenges include tumor microenvironment (TME) complexities, intra- and extra-tumoral molecular and biological heterogeneity, differences in metabolic and immune responses, and the presence of drug-resistant cancer stem cells capable of repopulating cancers subjected prior to therapies [151].

In this context, our efforts have focused on understanding the TME in breast cancer, using spontaneous, induced, or xenograft murine models of breast cancer [109,110,120,152] and spontaneous mammary tumors in dogs and cats [14,104,119,146,147,149].

Driven by our studies, collaborators, and others eliciting key molecular and cellular pathways associated with tumor progression in breast cancer, therapeutic studies were conducted to identify new treatment protocols using drugs already approved by international and federal regulatory bodies or to identify new compounds with antitumor activity.

Thalidomide is a drug with antiangiogenic, immunomodulatory, and anti-inflammatory properties that has been approved for the treatment of refractory multiple myeloma in humans, as well as other inflammatory diseases [149,153,154]. Our studies have demonstrated that thalidomide treatment promoted a reduction in metastatic tumor burden of the 4T1 tumor, associated with leukocytosis, decreased tumor inflammation and angiogenesis, a reduction in tumor-associated macrophages (TAMs) in the primary tumor, and an increase in these cells in metastasis-affected lungs [116,117,155]. However, in canine mammary carcinomas, the antitumor and anti-angiogenic effects of thalidomide were not observed [156]. Indeed, in a phase II clinical trial in humans, thalidomide showed little to no antitumor effect in metastatic breast cancer [157]. This highlights the limitations of translating therapeutic studies of this drug from xenograft murine models to human and veterinary medicine. Nevertheless, studies combining thalidomide with chemotherapy, particularly carboplatin, have proven effective in both the 4T1 murine model and canine mammary carcinomas [156].

In studies involving Ehrlich tumors, the impact of thyroid hormones on tumor growth was investigated in both solid and ascitic forms in adult female mice, both ovariectomized and non-ovariectomized. The findings indicated that hypothyroidism delayed tumor progression in both forms while maintaining cellular characteristics indicative of high proliferative potential and malignancy. Interestingly, ovariectomy alone caused only minor alterations, but when combined with hypothyroidism, it significantly enhanced the delay in tumor growth [105,158]. In contrast, hyperthyroidism led to a significant increase in tumor size in non-ovariectomized mice, with a less pronounced effect in ovariectomized mice [105].

The impact of natural extracts on tumor progression has also been a significant area of investigation. The aqueous extract of *Agaricus blazei* Murill was assessed for its effects on tumor growth and lymph node metastasis. While an initial reduction in tumor growth was noted, no significant differences were observed in relative tumor weight or proliferation rates between treated and untreated mice. Metastases were detected in the lymph nodes of all mice [159]. Similarly, ethanolic and aqueous extracts of *Arrabidaea chica* demonstrated efficacy in reducing tumor progression following oral administration, with notable effects on hematological profiles, including neutrophil counts, globulin levels, and the proportions of CD4+ and CD8+ lymphocytes [59]. These findings underline the critical role of inflammation in the progression of Ehrlich tumors in mice.

Other systemic factors, such as high-sugar and high-fat diets, also play a significant role in the pathogenesis of breast cancer. Diets rich in sugar and fat have been shown to influence immune responses and promote tumor growth in a murine model of 7,12-dimethylbenz[a]anthracene (DMBA)/medroxyprogesterone (MPA)-induced breast cancer [60] and in the transplantable 4T1 cell model [160]. The use of a selective cyclooxygenase-2 (COX-2) inhibitor, etoricoxib, reduced various inflammatory and pro-angiogenic mediators, resulting in decreased tumor growth and increased survival in animals with mammary tumors treated with a diet rich in sugar/fat [75].

Tumor inhibition by ketoprofen, a nonsteroidal anti-inflammatory drug, was also observed in the Ehrlich solid tumor model; however, no differences were found in the percentage of inflammation, hemorrhage, or tumor necrosis [161]. In humans, 93.5% of the breast cancer patients in our study exhibited high body fat and deficiencies in calcium and vitamin A, B6, and B12 intake [162]. Among human breast cancer patients, obesity was also associated with a higher risk of cancer recurrence and decreased survival [163].

Beyond conventional drug delivery methods, nanosystem-based therapies have been investigated to achieve more effective treatments with fewer side effects. For instance, a nanostructured lipid carrier (NLC) loaded with doxorubicin (DOX) demonstrated superior tumor reduction in the 4T1 breast cancer model compared to liposomes or free DOX [164]. Moreover, combining NLC-DOX with docosahexaenoic acid (DHA) proved effective not only for treatment but also for diagnostic and monitoring applications, broadening its potential utility in breast cancer therapy [165]. Similarly, formulations such as liposomes loaded with DOX and alpha-tocopheryl succinate, pH-sensitive liposomes containing ursolic acid, and liposomes co-encapsulating paclitaxel and doxorubicin enhanced antitumor efficacy while simultaneously reducing systemic toxicity [166,167,168].

Between 2020 and 2024, several innovative nanosystem platforms were developed, including exosome-fused liposomes loaded with doxorubicin (ExoSpHL-DOX), pH-sensitive PEGylated liposomes carrying cisplatin or doxorubicin (pHPL-CIS/DOX), and hybrid PEGylated pH-sensitive liposomes combined with extracellular vesicles derived from human breast cancer cells (MDA-MB-231) [59,167,169].

In studies using ExoSpHL-DOX, 4T1 tumor-bearing mice demonstrated increased cellular uptake of the formulation by 4T1 cells, significant tumor growth reduction, and decreased mortality rates. Importantly, this platform also exhibited substantially reduced toxicity to the liver and heart, revealing its potential for breast cancer therapy [169]. Furthermore, non-PEGylated liposomes were shown to achieve superior tumor targeting compared to their PEGylated counterparts, providing critical insights into the optimization of nanosystem design for enhanced therapeutic efficacy [59,167]. Finally, the hybrid pegylated pH-sensitive liposome-extracellular vesicle derived from the MDA-MB-231 cell line showed cytotoxicity against human breast cancer cell lines with different molecular subtypes, enhanced anti-migration properties, and exhibited a similar cellular uptake to the free DOX treatment. Yet, preliminary acute toxicity assessments using BALB/c female mice indicated a median lethal dose of 15–17.5 mg/kg, with no evidence of splenic, liver, heart, bone marrow, or renal cytotoxicity at a dose of 15 mg/kg [170].

## 7. Treatment (Canine and Feline)

Despite the absence of robust prospective clinical trial data allowing us to confidently determine the best treatment strategies for canine and feline mammary gland neoplasms [171], our group has made “significant” contributions to the field with multiple studies that evaluate surgical and systemic therapies.

In addition to enabling histopathological assessment, surgical excision increases overall survival and quality of life and may be curative in non-metastatic cases [55]. Adjuvant therapies are commonly recommended for high-risk mammary neoplasms, i.e., large tumors, advanced clinical staging, aggressive histology, and histological grade [54,171]. Indeed, Nunes et al. (2019) [52] demonstrated that carcinosarcomas clearly benefited from the addition of adjuvant therapies (chemotherapy with or without anti-angiogenic agents) following surgical excision, resulting in a more than three-fold increase in median overall survival. Importantly, the benefit of adjuvant therapies was not shown in carcinomas in mixed tumors, a tumor type associated with a superior prognosis [52].

In our evaluation of chemotherapy and cyclooxygenase (COX) inhibitors as adjuncts to surgery, we found a low COX-2 score in 41% and high COX-2 score in 59% of the evaluated cases. An increased COX-2 score was associated with decreased overall survival. Importantly, adjuvant chemotherapy with carboplatin, with or without COX-2 inhibitors (piroxicam or firocoxib), significantly improved overall survival compared to surgery alone, with minimal adverse events [172].

Then, we evaluated the addition of anti-angiogenic and immunomodulatory therapies to surgical resection and carboplatin-based chemotherapy [157]. The addition of thalidomide or metronomic chemotherapy with cyclophosphamide and firocoxib more than doubled the median overall survival in dogs with distant metastases compared to surgery alone or surgery with carboplatin. Remarkably, median overall survival exceeded one year in these advanced-stage cases, with many patients experiencing slower disease progression and, in some cases, regression of distant metastatic lesions, leading to markedly improved clinical outcomes.

Thalidomide’s safety profile was also evaluated in dogs with advanced-clinical-stage mammary gland neoplasms. The drug was well-tolerated, with minor alterations in complete blood count and serum biochemistry. While 50% of patients experienced increased somnolence at a dose of 20 mg/kg, less than 10% of pet owners reported a negative impact on the animal’s quality of life [57].

Collectively, our findings strongly support the use of adjuvant therapies for high-risk canine mammary gland neoplasms. These treatments were well-tolerated and resulted in significant survival benefits in patients with advanced clinical staging and aggressive histological features.

It is important to continue to explore additional treatment protocols, dissecting therapeutic response by high-risk features.

Finally, we conducted a retrospective study on the impact of adjuvant chemotherapy with carboplatin in feline mammary gland neoplasms with an advanced clinical stage and high histological grade. Although carboplatin was well-tolerated, no significant survival benefit was observed [173]. Further studies are needed to identify effective adjuvant treatments for feline mammary neoplasms.

There are ongoing controversies regarding the treatment of mammary neoplasms in female dogs and cats. Chemotherapy protocols remain inadequately established due to the need for standardization and uniformity in staging criteria and, most importantly, histopathological diagnosis. This highlights the importance of adopting the same diagnostic criteria used in human oncology to achieve translational objectives [34]. Moreover, the similarities between human and canine neoplasms extend to responses to conventional therapies, the development of treatment resistance, tumor dissemination during metastasis, and recurrence patterns. These interspecies similarities have enabled the application of targeted therapy knowledge from human oncology to animals with comparable molecular profiles. Despite the limited data and understanding of animal tumors, the advancement of precision medicine in veterinary oncology has been facilitated by these characteristics shared among the species investigated [174].

## 8. Conclusions and Future Directions

In vivo breast cancer models, whether experimental or spontaneous, have demonstrated broad utility in various contexts and will continue to play a pivotal role in understanding tumor progression mechanisms, therapeutic responses, and treatment resistance. Over the past decades, this field has advanced significantly, and it is poised to evolve further with the development of new tools that expand the available arsenal for investigating unresolved scientific questions. However, it is imperative for researchers to consider the inherent limitations of each model and carefully select the one that best represents the specific biological processes under study, aligning with the unique objectives of the research.

Furthermore, fostering greater interdisciplinary collaboration and translational research among the fields of veterinary oncology, medical oncology, and other cancer research areas is crucial. This includes engaging professionals such as biomedical scientists, biologists, pharmacists, nutritionists, and nurses, promoting the active contribution of each field. Mammary cancer remains the leading cause of cancer-related mortality among women worldwide and also exhibits a high prevalence in female dogs and cats. So far, the group’s study focus has been on comparative morphological aspects of spontaneous mammary neoplasms and prognostic and predictive factors; however, genetic studies may in the future complement the data obtained to date.

It is important to recognize that there is no single ideal experimental model. Breast cancer is highly heterogeneous in its histopathology, growth factor dependence, activation or inactivation of specific genes, clinical progression, and treatment response. Thus, a detailed understanding of the characteristics and limitations of the selected model is essential to maximize its scientific relevance.

## Figures and Tables

**Table 1:** Histological types described in women and identified in female dogs and/or cats based on the WHO classification [21] and the Consensus Regarding the Diagnosis, Prognosis, and Treatment of Canine Mammary Tumors, for dogs and cats [14,16,32,34].

		Woman	Dog	Cat
**BENIGN**	Ductal adenoma	x	x	x
	Tubular adenoma	x	x	x
	Lactating adenoma	x	x	x
	Ductal papilloma	x	x	x
	Adenomyoepithelioma	x	x	x
	Fibroadenoma	x	x	x
	Benign phyllodes tumor	x	x	x
	Pleomorphic adenoma/benign mixed tumor	x	x	x
**MALIGNANT**	Invasive carcinoma of no special type	x	x *	-
	Ductal carcinoma in situ	x	x	x
	Tubular carcinoma	x	x	x
	Invasive papillary carcinoma	x	x	x
	Solid papillary carcinoma	x	x	x
	Cribriform carcinoma	x	x	x
	Invasive micropapillary carcinoma	x	x	x
	Pleomorphic lobular carcinoma	x	x	-
	Secretory carcinoma	x	x	x
	Metaplastic carcinoma/carcinoma in a mixed tumor	x	x	x
	Malignant adenomyoepithelioma	x	x	x
	Mucinous carcinoma	x	x	x
	Neuroendocrine carcinoma	x	x	-
	Carcinoma with apocrine differentiation	x	x	x

* Solid-pattern carcinoma is equivalent to an invasive carcinoma of no special type with histological grade III. x = Histological type found in this species. - = Histological type not found in this species.

**Table 2:** Prognostic and predictive factors for canine, feline, and human mammary neoplasms [54,55,62].

Factors	Woman	Dog	Cat
**PROGNOSTIC**	Clinical staging (TNM)	Clinical staging (TNM)	Clinical staging (TNM)
	Tumoral size	Tumoral size	Tumoral size
	Lymph node metastasis	Lymph node metastasis	Lymph node metastasis
	Histological type	Histological type	Histological type
	Histological grading	Histological grading	Histological grading
	Hormonal receptors (ER, PR*)	Hormonal receptors (ER, PR*)	-
	Ki67	Ki67	Ki67
	HER-2	-	HER-2
	Molecular profile	-	-
	-	Cox-2	Cox-2
**PREDICTIVE**	Hormonal receptors (ER, PR*)	Hormonal receptors (ER, PR*)	Hormonal receptors (ER, PR*)
	Ki67	Ki67	Ki67
	Her-2	Cox-2	Cox2

ER: estrogen receptor; PR*: progesterone receptor. Further studies with larger sample sizes are necessary.

**Table 3:** Immunophenotypic classification of mammary neoplasms in dogs [41].

	Luminal A	Luminal B		HER-2 Overexpressed	Triple Negative/Basal Like
		HER-2 −	HER-2 +		
ER	+ e/ou PR+	+ e/ou PR+	+ e/ou PR+	-	-
PR	+ e/ou ER+	+ e/ou ER+	+ e/ou ER+	-	-
HER-2	−	-	+	+	-
Ki-67	<20%	≥20%	AS	AS	AS

ER: estrogen receptor; PR: progesterone receptor; AS: any score.

## Data Availability

No new data were created or analyzed in this study. Data sharing is not applicable to this article.
